# Extracellular Vesicles: A Potential Novel Regulator of Obesity and Its Associated Complications

**DOI:** 10.3390/children5110152

**Published:** 2018-11-15

**Authors:** Ahlee Kim, Amy S. Shah, Takahisa Nakamura

**Affiliations:** 1 Division of Endocrinology, Cincinnati Children’s Hospital Medical Center, 3333 Burnet Ave., Cincinnati, OH 45229, USA; Ahlee.Kim@cchmc.org; 2 Department of Pediatrics, University of Cincinnati College of Medicine, 3333 Burnet Ave., Cincinnati, OH 45229, USA

**Keywords:** type 2 diabetes, obesity, extracellular vesicles, exosomes, pediatrics

## Abstract

Childhood obesity continues to be a major public health concern. Obesity causes various metabolic complications, including insulin resistance, type 2 diabetes mellitus (T2DM), non-alcoholic fatty liver disease (NAFLD), dyslipidemia, and cardiovascular disease. However, currently, we have a limited understanding of the pathophysiology in the development of these processes. Extracellular vesicles (EVs) are nano-sized vesicles secreted by different cell types that travel to various organ systems carrying molecular and genetic information. These vesicles have been proposed as a novel intercellular communication mode in systemic metabolic regulation and in several pathophysiologic processes. In particular, recent studies indicate that EVs play a critical role in the pathogenesis of obesity and its metabolic complications. In this study, we reviewed the current literature that supports the role of EVs in the regulation of metabolic homeostasis and pathogenesis of obesity and its associated metabolic complications, with a short discussion about future directions in the EV research field.

## 1. Introduction

Recent data from the Centers for Disease Control and Prevention (CDC) demonstrate that more than one-third of the pediatric population is overweight or obese, and it is predicted that more than half of today’s youth will be obese by the time they reach mid-adulthood [1]. In parallel, the prevalence of obesity-associated comorbidities, including insulin resistance, type 2 diabetes mellitus (T2DM), dyslipidemia, non-alcoholic fatty liver disease (NAFLD) and cardiovascular disease have increased at an alarming rate [2,3,4]. While obesity and its associated complications are rising [5], we still lack a complete understanding of mechanisms by which these processes develop. One emerging area of research is the role of extracellular vesicles (EVs) in metabolic diseases largely because EVs have been shown to serve as a mode of intercellular communication in the regulation of metabolic processes, including inflammation, pancreatic beta (β) cell function, insulin sensitivity, and lipid metabolism.

**Figure 1:**
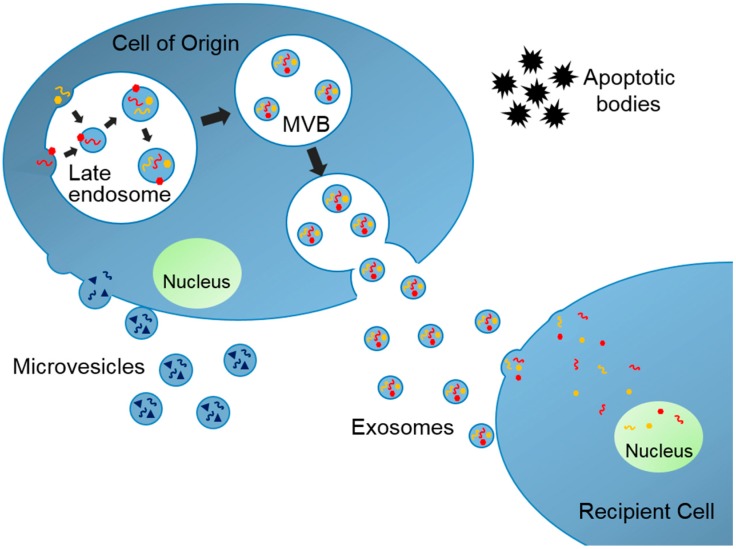
Biogenesis of Extracellular Vesicles. Exosomes are assembled within multivesicular bodies (MVBs) where specific exosomal cargos are sorted into exosomes. Microvesicles are generated from plasma membrane budding. Apoptotic bodies are aggregates of plasma membrane bleb. These vesicles travel to neighboring and distant organs and change the cellular function at the recipient tissues.

EVs are produced in their cells of origin and secreted to extracellular space where they travel to neighboring or distant organs (Figure 1). There are mainly three subtypes of EVs and each subtype has unique cellular biogenesis and characteristics [6]. Exosomes are nano-sized (30–150 nm in diameter) membrane-enclosed extracellular vesicles originating from intracellular organelles, multivesicular bodies (MVBs). Microvesicles are rather heterogeneously sized (100–1000 nm in diameter), and they are produced by budding of plasma membrane. Finally, apoptotic bodies are aggregates of plasma membrane bleb during apoptosis and these bodies also have heterogeneous size (50–5000 nm). Exosomes along with microvesicles and apoptotic bodies are not always distinguishable because they share many similar molecular characteristics but are distinct entities with different cellular functions. EVs contain various cargos from their cells of origin, including lipids, proteins, and nucleic acids, such as mRNAs, tRNAs, and microRNAs (miRNAs, small noncoding RNAs regulating gene expression that suppress specific mRNAs’ translation). EVs function as vectors of this biological information and can modify the cellular function of the recipient organs. Therefore, the EVs have been termed “a novel mechanism for intercellular communication” [7]. As a result, the EV research field has exploded with studies suggesting potential roles of those vesicles as a prognostic factor, a biomarker, and a therapeutic target for a number of diseases, including cancer, infectious disease, neurologic disease, and metabolic diseases [6,8,9]. We will review key literature where EVs have been identified and associated with the pathogenesis of obesity and development of metabolic complications including T2DM and NAFLD. We conclude with remarks on much-needed areas of research specifically in pediatric obesity and metabolic diseases.

## 2. EVs and Obesity

Recent data indicate that EVs play a role in obesity and development of its metabolic complications by serving as a mode of intercellular communication among adipose tissue, liver, skeletal muscle, and immune cells [6]. Studies have shown obesity is associated with an increase in the number of circulating EVs. Stepanian et al. reported women with severe obesity had a higher number of plasma EV compared to a normal-weight control group [10]. Subsequently, Ferrante et al. identified 55 adipocyte-derived exosomal miRNAs that were differentially expressed between obese and lean individuals, including an upregulation of miR-23b and miR-4419 and a downregulation of miR-148b and miR-4269, suggesting exosomal miRNAs were associated with metabolic changes in obese individuals [11]. In human studies, a strong correlation between the number of circulating exosomes and maternal BMI has been observed [12]. Importantly, it was also reported that the adipocyte-derived exosomal miRNA profile was modified 1 year following gastric bypass surgery [13]. Following surgery, ten exosomal miRNAs involved in the insulin signaling pathway were different and their levels correlated with changes in Homeostasis model assessment (HOMA) pre- and post-surgically. These results suggested an association between exosomes and the improvement in insulin sensitivity post-bariatric surgery via transferring exosomal miRNAs between tissues.

One proposed mechanism by which obesity leads to various metabolic complications is via inducing inflammation in major metabolic organs, including the liver, adipose tissue, and skeletal muscle [14]. Chronic low-grade systemic inflammation is a crucial clinical characteristic of obesity, and recent data suggests that inflammation plays a pivotal role in the development of obesity-induced metabolic complications, such as T2DM, NAFLD, and cardiovascular disease [14,15].

Current data propose that EVs function in metabolic regulation via modulating immune function. Deng et al. reported that exosomes released from adipocytes stimulated the differentiation of monocytes into active macrophages and induced increased pro-inflammatory cytokine secretion, such as IL-1 and TNF-α [16]. Kranendonk et al. found that the pro-inflammatory effect of adipocyte-derived exosomes was more pronounced when the vesicles were released from visceral adipose tissue (VAT) compared to subcutaneous adipose tissue, and when these vesicles contained adiponectin, a marker for adipocyte origin as opposed to non-adipocyte cells in adipose tissue [17]. Freeman et al. also identified that exosomes from patients with diabetes induced increased levels of cytokine secretion from human monocytes [18]. These studies suggested the pro-inflammatory roles of exosomes. However, investigators have demonstrated that exosomes also have anti-inflammatory effects. Song et al. found that adipose-derived stem cells secreted exosomes that attenuated adipose inflammation by driving M2 polarization of adipose tissue macrophages (ATM), and subsequently preventing diabetic conditions, hepatic steatosis, and obesity progression [19].

These pro- or anti-inflammatory immune modulating functions of exosomes were proposed to be mediated by specific exosome cargos, such as miRNAs or proteins. Ferrante et al. identified exosomal-miRNAs that were differentially expressed between lean and obese individuals, modulate growth factor-beta (TGF-β) signaling, a pro-inflammatory cytokine [11]. Alexander et al. described that the exosomes carrying distinct miRNAs can have a different function on immune modulation [20,21]. For examples, exosomes secreted from primary bone marrow-derived dendritic cells carrying miR-146a or miR-155 can either inhibit or promote an endotoxin-induced inflammatory response of recipient cells both in vitro and in vivo [21]. Similarly, exosomes without the presence of miR-155 do not have the same pro-inflammatory function as the exosomes with the presence of miR-155 [20]. Data suggest that exosomal proteins also function in determining specific exosomal functions in the modulation of immune function. Huang et al. proposed that it was the specific immunoglobulins transferred within exosomes, that activated complement pathways [22], and Zhao et al. proposed that exosome function on M2 macrophage polarization was mediated by one of the exosomal proteins, Signal transducer and activator of transcription 3 (STAT3) [19].

## 3. EVs and Insulin Resistance/Type 2 Diabetes

A complex communication has been described between the pancreatic β cell and insulin-responsive organs, such as liver, adipocytes, and skeletal muscle to maintain normal glucose metabolism. Exosomes have been linked to the pathophysiology of diabetes by serving as a mode of intercellular communication among major metabolic organs, including the liver, adipose tissue, skeletal muscle, and β cells. In 2002, Sabatier et al. reported that patients with type 1 and type 2 diabetes had increased levels of circulating EVs compared to controls [23]. Similarly, Freeman et al. reported a higher level of plasma EVs in patients with diabetes compared to controls [18], and a consistent trend was also reported in diabetic mice that exhibited a higher number of plasma exosomes compared to control mice [22]. Most recently, Kobayashi et al. demonstrated that individuals with impaired oral glucose tolerance test (OGTT) had a significantly higher number of circulating EVs compared to those with normal OGTT and the number of circulating EVs correlated with Homeostasis model assessment β-cell function (HOMA-β) [24]. Accumulating evidence suggests that EVs play a direct role in regulating β-cell function in the pathogenesis of T2DM [25,26]. Jalabert et al. demonstrated that the exosomes secreted from skeletal muscles of high-fat diet (HFD) mice induced proliferation of pancreatic cells, whereas skeletal muscle-derived exosomes from regular diet mice did not [27]. Cantaloppi et al. showed that microvesicles derived from endothelial progenitor cells (EPCs) enhanced human pancreatic islet vascularization, and this effect was ameliorated with microvesicles pretreated with RNase or derived from EPCs when Dicer, a core enzyme of miRNA biogenesis, was knocked-down, suggesting that the effects of microvesicles were through their miRNA cargos [28].

Pancreatic β cells also secrete EVs carrying specific β cell markers, such as insulin transcripts [29,30]. β cells appear to interact with neighboring β cells through a communication mediated by EVs. Guay et al. reported that the β cell-derived exosomes affected the survival of neighboring β cells by transferring miRNAs [30]. More recently, Ribeiro et al. reported that the exosomes secreted from healthy pancreatic β cells had the ability to suppress aggregation of islet amyloid polypeptide (IAPP) in neighboring β cells, whereas exosomes secreted from β cells of the T2DM pancreas failed to do this, mimicking the pathophysiology of T2DM [31]. These findings complemented the pre-existing hypothesis that islet-derived microRNAs function in the regulation of β cells’ functions in a paracrine manner, and the islet microRNAs differed between the healthy pancreas and T2DM pancreas [32].

Exosomes may also play a role in the regulation of peripheral insulin sensitivity, a major component of the pathogenesis of T2DM. The number of circulating EVs or adipocyte-derived EVs have been positively correlated with Homeostasis model assessment insulin resistance index (HOMA-IR) [17,18], and distinctive EV cargos have been associated with regulation of insulin sensitivity [13,18]. Exosomes appeared to regulate insulin sensitivity through at least two different mechanisms, i.e., by modulating inflammation, as briefly introduced above [16,17,18], or by a direct interaction with insulin-responsive organs. The latter may occur directly or indirectly by affecting insulin signaling pathways. Kranendonk et al. proposed that human adipose tissue-derived EVs regulated hepatic and skeletal muscle insulin signaling through interaction with major insulin signaling molecules, such as serine/threonine kinase Akt [33], and Wang et al. demonstrated that exosomes derived from pancreatic cancer cells inhibited Phosphatidylinositol-3-kinase (PI3K)/Akt signaling pathway in muscle cells [34].

Data supporting an indirect effect on insulin signaling comes from Thomou et al. who generated mice lacking Dicer specifically in adipose tissue (these mice are called ADicerKO mice from here) [35]. These mice developed several metabolic phenotypes, including insulin resistance and glucose intolerance that improved with transplantation of fat from normal mice. Subsequently, the authors identified that miR-99b carried by adipocyte-derived exosomes modulated hepatic fibroblast growth factor 21 (FGF-21), and this pathway was proposed to be the mechanism of how adipocyte-derived exosomes regulated insulin sensitivity. In the same year, Ying et al. demonstrated that exosomes from the ATM of obese mice caused insulin resistance when the exosomes were transplanted to lean mice, while exosomes from the ATM of lean mice improved insulin resistance when transplanted to obese mice [36]. Further investigation identified a set of miRNAs that were differentially expressed in obese versus lean ATM-derived exosomes. miR-155, a microRNA regulating cellular insulin sensitivity by targeting peroxisome proliferator-activated receptor gamma (PPARγ) in adipocytes and hepatocytes showed a ~3-fold greater expression level in obese ATM-derived exosomes.

## 4. EVs and Non-Alcoholic Fatty Liver Disease

The pathogenesis of NAFLD/Nonalcoholic steatohepatitis (NASH) involves a complex intercellular communication to induce inflammation and fibrosis in the liver, and the current data support the role of EVs in this process. The liver secretes distinct EVs and also responds to exosomes/microvesicles secreted from other tissues or the liver itself. It appears several different organs send “pro-inflammatory/pro-fibrotic” signals to the liver via EVs. Korneck et al. observed that T cell-derived EVs regulated hepatic fibrosis [37], and Koeck et al. reported that exosomes from the VAT of obese individuals induced dysregulation of TGF-β pathway, a pathway associated with hepatic fibrosis [38]. Several studies reported that EVs also regulate hepatic fibrosis in a paracrine manner. Hepatocytes communicate with hepatic stellate cells via utilizing the EV system, and lipotoxicity on hepatocytes appears to alter the signal into a more fibrogenic signal [39,40]. A horizontal communication of the fibrotic signal between stellate cells, and a communication between hepatic myofibroblasts and hepatic endothelial cells also takes place via EVs [41]. Santangelo et al. identified one of the molecular mechanisms of miRNA sorting of hepatic exosomes [42], suggesting a novel therapeutic target for NAFLD/NASH. Kornek et al. described that patients with NAFLD/NASH had an increased number of circulating EVs derived from invariant natural killer cells and macrophages/monocytes compared to individuals without NAFLD/NASH [43]. The recent discovery of the distinctive pattern of EVs and their cargo associated with NAFLD/NASH may pave the way for the development of a novel biomarker for these conditions [43,44,45].

## 5. Conclusions

Accumulating data in animals and adults suggests that EVs are an important mediator in obesity and its associated metabolic complications. However, despite the explosion of studies in the last decade, challenges still exist. First, there are still technical difficulties in isolating and quantifying EVs from human plasma because the isolation is labor intensive and low yield. Second, there is an incomplete technique to identify specific tissue origins for EVs in vivo. Finally, reproducible experimental protocols to test the function of EVs both in vivo and in vitro are only just emerging. However, novel measures to isolate EVs in a more subgroup-specific way, such as an affinity-based exosome isolation technique are being developed [46]. Furthermore, EVs appear to be quite stable at −80 °C, which opens the possibility to utilizing newly developed techniques in the future.

Despite emerging evidence in adults, little is known about EVs in the pediatric population. Adolescents offer a unique population to study, because they are metabolically stressed for a relatively shorter time and metabolic complications in adolescents appear to be reversible at least with dramatic weight loss or surgery [47]. Additionally, adolescents exhibit fewer confounding variables, including medications (i.e., statins) and lifestyle choices (alcohol and smoking) which may influence inflammation. Therefore, studies in adolescents offer a novel opportunity to study the effects of obesity on EVs, but also on how EVs are influenced by diet and physical activity, and importantly whether EVs can be modified with interventions including weight loss and medical therapies that treat obesity and diabetes.
